# Prophylactic Anti-Cytomegalovirus Hyperimmunoglobulin in Critically Ill Liver Transplant Patients: Impact on Early Immunology and Survival

**DOI:** 10.3390/jcm9030656

**Published:** 2020-02-29

**Authors:** Arno Kornberg, Ulrike Witt, Jennifer Kornberg, Katharina Müller, Helmut Friess, Katharina Thrum

**Affiliations:** 1 School of Medicine, Department of Surgery, Klinikum rechts der Isar, Technical University of Munich, 81675 Munich, Germany; Ulrike.Witt@tum.de (U.W.); Helmut.Friess@tum.de (H.F.); 2 Department of Anaesthesiology, Klinikum Großhadern, Ludwig-Maximilian-University of Munich, 81377 Munich, Germany; JenniferKornberg@aol.com; 3 Department of Surgery, Friedrich-Schiller-University of Jena, 07747 Jena, Germany; katharina.mueller86@gmx.de; 4 Institute of Pathology, Helios Klinikum Berlin, 14165 Berlin, Germany; katharina.thrum@gmail.com

**Keywords:** liver transplantation, critical illness, multiorgan failure, CMV infection, anti-CMV hyperimmunoglobulin, immune modulation

## Abstract

Background: Anti-cytomegalovirus hyperimmunoglobulin (CMVIg) was shown to provide beneficial immunodulatory properties beyond antiviral efficacies. The aim of this retrospective study was to assess the impact of prophylactic CMVIg treatment on early outcome following liver transplantation (LT) in critically ill patients. Methods: Forty-three cirrhotic patients requiring pre-LT intensive care due to multiorgan failure were analyzed. Twenty-eight patients with enhanced CMV risk (D+/R+; D+/R−; D−/R+) received prophylactic CMVIg for a minimum of 7 days, while 15 patients (D−/R−) did not. Results: Post-transplantation rates of intra-abdominal infections (28% vs. 61.1%; *p* = 0.03), Epstein–Barr virus infections (0% vs. 33.3%; *p* = 0.034), allograft rejections (0% vs. 22.2%; *p* = 0.013) and sepsis-related mortality (4% vs. 27.8%; *p* = 0.026) were significantly lower, whereas incidence of CMV infections (4% vs. 22.2%; *p* = 0.066) tended to be lower in the CMVIg subset. In multivariate analysis, only pretransplant elevated serum lactate level (hazard ratio = 34.63; *p* = 0.009) and absence of CMVIg therapy (hazard ratio = 21.76; *p* = 0.023) were identified as independent promoters of 3-month mortality. Conclusion: Prophylactic treatment with CMVIg reduces predisposition for severe immunological and septic events and, thereby, early mortality in critically ill liver recipients.

## 1. Introduction

The implementation of the “sickest first” allocation policy using the so-called Model of End-Stage Liver Disease (MELD) score almost 2 decades ago has clearly proven effective in reducing waiting list mortality in many large liver transplantation (LT) programs around the world [1]. Due to a growing disparity between demand and availability of appropriate donor organs, pre-LT waiting times and final MELD scores have been, however, significantly increasing in recent years; consequently, transplant patients have become sicker and more complex [1,2]. In particular, multiorgan failure (MOF) requiring intensive care unit (ICU) support, as is common in patients with acute-on-chronic liver failure (ACLF), was shown to be a major risk factor of early morbidity and mortality following LT [3,4,5]. Considering other established transplant indications that are associated with a much better prognosis, LT in ICU-bound patients not only represents a medical challenge but also raises important ethical issues [6]. In order to avoid futility in patients being “too sick” for transplant, a profound interdisciplinary risk assessment using reliable prognostic factors is mandatory [7]. Nonetheless, owing to an enhanced susceptibility to severe immunological and septic complications, the postoperative course in this specific LT-subset remains highly demanding [3,4,5], thus requiring the implementation of effective immunoprotective concepts beyond adjustment of immunosuppressive treatment [8,9].

In this context, prevention of early cytomegalovirus (CMV) infection seems to be of particular prognostic importance, since besides causing direct organ damages, it may indirectly affect outcome by modulating the recipients’ immune response. Triggered by proinflammatory cytokine release and various immunosuppressive mechanisms, an immunological risk may, thereby, be aggravated [10]. Although antiviral prophylaxis by ganciclovir or valganciclovir is nowadays widely established in patients with high-risk CMV immunoglobulin (Ig) G donor (D)/recipient (R) seroconstellation (D+/R−), this is usually not applicable in severely immunocompromised allograft recipients due to intolerance against common side effects like renal dysfunction, leukopenia and thrombocytopenia [11]. That is why anti-CMV hyperimmunoglobulin (CMVIg) for passive immunization is meanwhile increasingly reconsidered in selected patients [12]. Although tolerability and antiviral efficacies of CMVIg have clearly been confirmed in the past [13], its use is currently not a recommended standard for antiviral prophylaxis or treatment in the LT setting [14]. In recent years, there seems to be growing suggestive evidence that, apart from antiviral activities, CMVIg may provide anti-inflammatory and immunoregulatory properties that could be valuable to counteract CMV-related immune reactions [12,15,16,17]. We hypothesized that critically ill liver recipients, who are particularly threatened by early inflammatory events, might benefit from these positive immunomodulatory capabilities. The aim of the presented retrospective study was to assess the prognostic impact of prophylactic CMVIg therapy on early outcome in a series of highest acuity ICU-bound LT patients.

## 2. Material and Methods

### 2.1. Study Cohort

From a prospectively managed LT database, 43 adult cirrhotic patients with hepatic decompensation requiring ICU support prior to LT were identified between 2007 and 2012. At the time of listing, all of them had expressed their consent that data may be used for scientific purpose. The study was conducted in accordance with the Declaration of Helsinki, and the protocol was approved by the institutional review board of the Medical School of the Technical University of Munich (No. 217/15). It is noteworthy that no organs from executed donors were used in this investigation. Progressive liver cirrhosis was assessed by clinical and radiographic criteria prior to registration and could be confirmed on histopathological analysis of the explanted livers. Patients with acute liver failure were not included in the study. In addition to liver dysfunction, all patients suffered from associated neurologic, hemodynamic, respiratory or renal failure requiring mechanical invasive ventilation, continuous vasopressor administration or renal replacement therapy. “Fatal triad” indicated the necessity of pulmonary, circulatory and renal support. Laboratory (lab.) MELD scores at listing and at LT were used for describing MELD dynamics (ΔMELD score = (lab.) MELD score at registration – (lab.) MELD score at LT). Diagnosis and grading (grades 1–3) of ACLF and related MOF were based on the European Association for the Study of the Liver—Chronic Liver Failure (EASL-CLIF) Consortium consensus criteria. The respective CLIF-C organ failure (OF) score (range: 11–18) at LT was calculated for each patient [18]. Preoperative serum lactate levels (normal range: <1.8 mmol/L) were used for describing severity of critical illness and tissue hypoxia [19,20], whereas values of C-reactive protein (CRP; normal range: <0.5 mg/dL), interleukin-6 (IL-6; normal range: <7 pg/mL) and procalcitonin (PCT; normal range: <0.1 ng/mL) reflected extent of systemic inflammatory response reaction prior to LT [21].

### 2.2. Donor Factors

The following prognostically relevant donor factors were included in risk analysis: age, body mass index, donor risk index, liver allograft reperfusion performance, cold and warm ischemia times.

### 2.3. Treatment with CMVIg and Post-Transplant Follow-Up

According to our standard transplant protocol, ICU-bound liver recipients with an increased CMV risk (D+/R+; D+/R−; D−/R+) received prophylactic intravenous CMVIg (Cytotect™, Biotest, Dreieich, Germany) with a dose of 100 IE (Ehrlich)/kg/day (≙ 1mL/kg/day; infusion rate: 0.8 mL/kg/h), starting at the first post-LT day and continued for a minimum of 1 week, whereas D−/R− patients were not treated. As a result of MOF, none of the study patients were eligible for specific antiviral medication. Serum DNA loads of CMV and Epstein–Barr virus (EBV) were determined in all patients twice a week during post-LT ICU stay and weekly at minimum thereafter for the first 3 months by real-time PCR (ThermoFisher 7500 Real-Time PCR System, ThermoFisher Scientific Inc., USA). Detectable CMV DNAemia at any level indicated CMV infection. Immunosuppressive therapy consisted of a dual regimen by tacrolimus (Tac; intended trough level: 5–7 ng/dL) and corticosteroids. Biopsy-proven allograft rejection was treated with methylprednisolone bolus/Tac-dose adjustment. Peak serum levels of CRP, IL-6 and PCT during 1 month post-LT described early systemic state of inflammation [21].

### 2.4. Statistical Analysis

Quantitative variables were expressed in median and range and compared by ANOVA analysis. Qualitative variables were reported in frequencies and percentages and compared by chi-squared test. Post-LT survival rates were determined by Kaplan–Meier method and compared using log rank test. Factors of early (3-month) mortality were assessed by uni- and multivariate logistic regression analysis. A *p* < 0.05 was considered statistically significant. All statistical analyses were performed using SPSS 24.0 software (IBM Inc., Munich, Germany).

## 3. Results

### 3.1. Patients’ Characteristics

Median age of the study population was 58 years (range: 32–68), with ethyltoxic liver disease (58.1%) and viral hepatitis (23.3%) being the major transplant indications. Median (lab.) MELD scores at listing and at LT were 28 (range: 7–40) and 38 (range: 30–40), respectively, resulting in a median ΔMELD of 9 (range: 0–32). Pre-LT waiting times measured from registration and from final hospital admittance ranged between 4 days and 36 months (median: 3 months) and between 3 and 71 days (median: 15 days), respectively. All patients were ICU-bound prior to LT for a median of 6 days (range: 2–42). With regard to ACLF, patients were classified grade 1 in 2 (4.7%), grade 2 in 10 (23.3%), and grade 3 in 31 cases (72.1%). Twenty-five patients required dialysis (58.1%), 36 patients received vasopressors (83.7%), and 18 patients (41.9%) were on ventilation treatment prior to LT. Twenty-five liver recipients (58.1%) received prophylactic anti-CMV Ig for a median of 7 days (range: 7–20; Table 1). Recipients’ age was significantly lower and pre-LT CRP and IL-6 levels tended to be lower in the CMVIg subset, whereas in contrast, no significant differences were noticed regarding severity of liver disease and MOF. In addition, donor variables were comparable between both subsets (Table 1).

### 3.2. Early Post-LT Outcome

**Figure 1:**
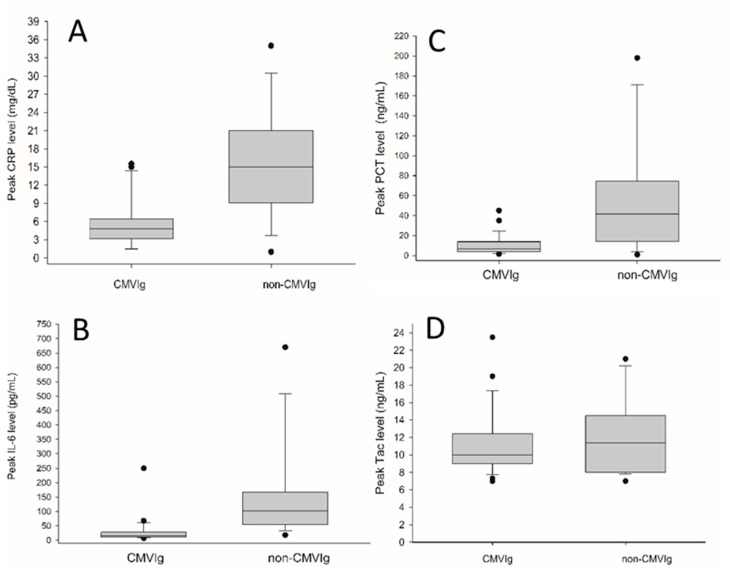
Post-LT peak serum levels of CRP (**A**), *p* < 0.001; IL-6 (**B**), *p* < 0.001 and PCT (**C**) *p* = 0.017 were all significantly lower following CMVIg treatment, whereas peak Tac levels (**D**) *p* = 0.336 were comparable.

Treatment was well-tolerated in all cases, without need of dose reduction. Median total number of CMVIg doses/patient was 7 (range: 7–20). The incidence of intra-abdominal infections was significantly higher in the non-CMVIg cohort, while rates of systemic bacterial infections were comparable (Table 2). One patient of the CMVIg- (4%) but four of the non-CMVIg subset (22.2%) developed CMV infection. Apart from that, incidence of EBV infections was significantly higher without therapy. Allograft rejection rates were 0% for the CMVIg cohort and 22.2% for the non-CMVIg cohort (Table 2). Post-transplant peak serum levels of CRP, IL-6 and PCT were all significantly lower following anti-CMV Ig treatment, whereas Tac levels were comparable (Figure 1).

### 3.3. Prognostic Factors of 3-Month Mortality

**Figure 2:**
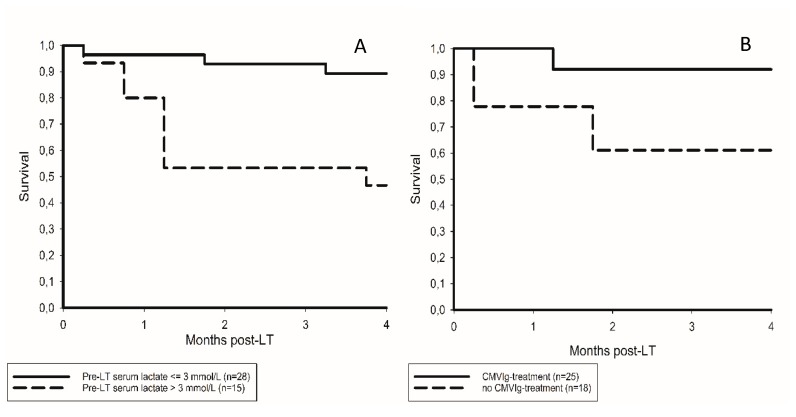
(**A**) Postoperative survival rates at 3 months were 92.9% and 53.3% in patients with pre-LT serum lactate levels ≤ versus >3 mmol/L, respectively, log rank = 0.002. (**B**) Survival rates in patients receiving and those not receiving CMVIg were 92% and 61.1%, respectively, log rank = 0.02.

At 3 months post-LT, nine liver recipients had died (20.9%), two in the CMVIg (8%) subgroup and seven in the non-CMVIg subgroup (38.9%). Corresponding mortality rates due to septic MOF were 4% and 27.8%, respectively (Table 2). In univariate analysis, MELD score at listing, ΔMELD, ICU stay, fatal triad, CMVIg treatment, cold ischemia time and serum levels of lactate, CRP and IL-6 exerted a prognostic impact (Table 3). Only pre-LT elevated lactate level and absence of CMVIg therapy were identified as independent promoters of 3-month mortality, whereas cold ischemia time almost reached statistical significance (Table 4). Respective survival curves are illustrated in Figure 2.

## 4. Discussion

The results of our study indicate that prophylactic administration of CMVIg reduces early morbidity and mortality following LT in critically ill cirrhotic patients, most probably related to positive immune modulation with regard to CMV risk, allograft immunogenicity and predisposition for septic events (Table 2).

Owing to evidenced antiviral efficacies and low adverse-effect profile, anti-CMV Ig has for a long time been the cornerstone in prophylaxis and treatment of CMV infection following solid organ transplantation. However, in the context of potent and cheaper antiviral drugs that have been implemented in the last years [11], it has clearly lost significance. Recent guidelines issued by the Transplantation Society do not generally recommend prophylaxis with CMVIg, but rather suggest augmentation of antiviral medication in high-risk organ recipients, who require intensified immunosuppression, such as patients following thoracic and intestinal transplantation. Apart from that, its administration may be considered on an individual basis as rescue treatment in severe CMV diseases or ganciclovir-resistant courses [14].

Currently, there is growing experimental and clinical evidence that, independent from established antiviral capacities, CMVIg provides immunological benefits that seem to be rather comparable to those of polyvalent Ig preparations [12,15,16]. Even though the complex mechanisms of immune modulation are not yet fully understood, it appears to affect several aspects of the innate and adaptive immune response, resulting in a combination of immunosuppressive (inhibition of dendritic maturation and suppression of T cells), immunostimulatory (passive anti-CMV immunization and increasing levels of naïve B cells) and anti-inflammatory (decreasing production of IL-2, interferon-y and IL-10) efficacies [12,15,16,17,22,23,24]. In fact, lower incidences of immunological and infectious complications following anti-CMV Ig therapy have been demonstrated in some recent studies on adult and pediatric LT [25,26,27,28]. In the largest investigation thus far on this topic presented several years ago, Fischer et al. reported on reduced risk of allograft loss and death in 2350 liver recipients receiving CMVIg, without, however, stratifying data on extent of liver decompensation and MOF [28].

To the best of our knowledge, we here present the first study analyzing the impact of prophylactic CMVIg in severely ill ICU-bound liver transplant patients. Apart from pre-LT serum lactate, which is an established prognostic factor in this clinical setting [19,20], only lack of anti-CMV Ig therapy was identified as an independent promoter of early mortality in our series (Table 4). According to our findings, different immunomodulatory aspects may be discussed as possible triggers for the observed prognosis improvement.

First, despite higher serological CMV risk, we noticed a clear trend of lower CMV incidence in the CMVIg subset (4% vs. 22.2%), which in view of well-known direct and indirect CMV-related harms could have been involved in superior outcome. Thus, our data emphasize on the prognostic importance of implementing passive CMV-specific immunity in those cases where recommended antiviral prophylaxis is not feasible [17]. Second, rates of acute allograft rejections (0% vs. 27.8%) and severe septic events (4% vs. 27.8%) were both significantly lower in our treatment group (Table 2), even though contradictory trends are frequently observed in clinical reality. Therefore, anti-CMV Ig appears to stabilize the immunological balance between allograft immunogenicity on the one hand and susceptibility for infections on the other, which may be fundamental for reducing early morbidity and mortality in immunocompromised liver recipients [29]. In this context, a higher rate of EBV reactivation (33% vs. 0%; Table 2) may be regarded as another indicator of immunological dysbalance in the non-CMVIg subgroup [30]. Third, significantly lower post-LT peak serum levels of proinflammatory mediators (Figure 1) suggested a beneficial impact on early systemic immunological response reaction [21].

Retrospective design and low sample size are limitations of our study and may well explain the high 95% confidence interval in our multivariate analysis (Table 4). Apart from that, the prognostic value of CMVIg in low risk patients (D−/R−) remained undefined. Also, nonconsidered differences in proinflammatory activation might have biased our results. In fact, the immunological performance may be influenced by (pre-LT) background inflammation, donor liver function and (post-LT) immunosuppressive treatment [30]. Apart from recipients’ age, which in turn may have had a prognostic impact in our analysis [3,4,5], we did not find significant differences in patients’ characteristics, donor features and immunosuppressive levels (Table 1). However, dynamic immune monitoring including repeat IgG level determinations and immunosuppressive drug exposure studies are required for a more precise immunological assessment. Finally, we cannot exclude that treatment with nonspecific Ig might have provided similar immunomodulatory efficacies than CMVIg. Recently, a beneficial efficacy of IgM-enriched Ig on hemodynamic stability and 30-day mortality was reported in a series of 21 patients suffering from post-LT vasoplegia requiring vasopressor treatment. However, there was no control group and, moreover, the study was lacking data on pre-LT status and CMV-specific outcome [31]. Thus, in our opinion, the combination of both anti-CMV impact and immunoregulatory properties renders CMVIg more attractive for critically ill liver recipients than conventional Ig preparations, which do not specifically inhibit viral activities. However, a comparative investigation is needed to further elucidate this issue.

Particular strengths of our investigation included a comprehensive generation of preoperative background and post-LT follow-up data and, above all, topicality of the subject and novelty of our findings. Although the “sickest first” policy has clearly defined and transparent rules and regulations [32], decision-making process in times of a dramatic donor shortage is determined not only by considerations of medical feasibility, but also, to a large extent, by ethical concerns. Individual urgency and possible benefit in highly complex LT candidates have to be weighed against common utility [6], which is hampered by lack of well-defined drop-out criteria and, besides, by an enormous pressure to succeed with regard to survival rates and profitability of the program [6,7]. Instead of defining transplant futility [7,20,33], we were rather focusing on immunological aspects to improve survival of highest acuity LT patients who most probably had been rejected at utility-guided centers. Against this background, our reported 3-month survival rate of 92% following anti-CMV Ig treatment represents an extraordinary outcome result, especially when considering that death would have been the most likely alternative.

## 5. Conclusions

In summary, our study suggests that critically ill liver recipients benefit from prophylactic CMVIg treatment by an improved risk profile for immunological and infectious complications and, thereby, from reduced early mortality. However, this needs to be validated in a prospective multicenter study approach, including immunological analyses for clarifying the underlying immunomodulatory mechanisms of action.

## Figures and Tables

**Table 1:** Pretransplant patients’ and donors’ characteristics.

Patients’ Characteristics	Non-CMVIg (*n* = 18)	CMVIg (*n* = 25)	*p* Value
Age	62 (39–68)	56 (32–67)	0.007
Male sex	12 (66.7%)	16 (64%)	0.856
**Genesis of Cirrhosis**			
*Ethyltoxic*	12 (66,6%)	13 (52%)	0.519
*Viral hepatitis*	4 (22.2%)	6 (24%)	
*Autoimmune*	1 (5.6%)	5 (20%)	
*Cryptogenic*	1 (5.6%)	0 (0%)	
*Other*	0 (0%)	1 (4%)	
**CMV Seroconstellation**			
*D−/R−*	18 (100%)	0 (0%)	<0.001
*D−/R+*	0 (0%)	7 (28%)	
*D+/R−*	0 (0%)	14 (56%)	
*D+/R+*	0 (0%)	4 (16%)	
Serum bilirubin level in mg/dL at LT	14 (4.8–31.9)	14 (6.9–58)	0.232
Serum creatinine level in mg/dL at LT	2.6 (0.5–3.8)	2.5 (1.4–5.5)	0.579
International normalized ratio at LT	2.3 (1.5–3.1)	2.5 (1.2–4.0)	0.186
Serum natrium level in mmol/L at LT	130 (125–145)	133 (119–147)	0.523
(lab.) MELD score at listing	25.5 (12–40)	30 (7–40)	0.486
(lab.) MELD score at LT	39 (31–40)	38 (30–40)	0.621
ΔMELD score	10 (0–28)	9 (0–32)	0.752
Waiting time from registration in months	3 (0.5–25)	3 (0.1–36)	0.642
Waiting time from final admittance in days	14.5 (3–41)	17 (3–71)	0.225
Ascites at LT	18 (100%)	23 (92%)	0.219
Bacterial infection at LT	10 (55.6%)	10 (40%)	0.313
Duration of pre-LT ICU stay in days	7.5 (0–35)	4 (0–42)	0.276
Ventilation at LT	9 (50%)	8 (32%)	0.234
Vasopressors at LT	15 (83.3%)	21 (84%)	0.953
Renal replacement therapy at LT	10 (55.5%)	15 (60%)	0.771
Fatal triad at LT	6 (33.3%)	6 (24%)	0.501
**Acute on Chronic Liver Failure at LT**			
*Grade 1*	0 (0%)	2 (8%)	0.452
*Grade 2*	5 (27.8%)	5 (20%)	
*Grade 3*	13 (72.2%)	18 (72%)	
**Organ Failures (According CLIF-C) at LT**			
*Liver*	11 (61.1%)	13 (52%)	0.553
*Kidney*	18 (100%)	23 (92%)	0.219
*Circulation*	15 (83.3%)	21 (84%)	0.935
*Respiration*	10 (55.6%)	9 (36%)	0.201
*Coagulation*	10 (55.6%)	14 (56%)	0.977
*Neurology*	9 (50%)	9 (36%)	0.359
Number of Organ Failures at LT			
≤ 3	7 (38.9%)	13 (52%)	0.395
> 3	11 (61.1%)	12 (48%)	
CLIF-C OF score at LT	16 (11–18)	15 (11–18)	0.460
Lactate level in mmol/L at LT	3.0 (0.9–10)	3 (0.9–5.8)	0.373
CRP level in mg/dL at LT	3.8 (0.7–6.1)	3 (0.4–7.2)	0.058
PCT level in ng/mL at LT	2.5 (0.4–7)	1.3 (0.3–49.9)	0.480
IL-6 level in pg/mL at LT	22 (6–78)	17 (5–45)	0.074
**Donor Variables**			
Age	54 (18–75)	57 (29–71)	0.428
Body mass index	1.8 (1.1–2.5)	1.9 (1.3–2.3)	0.399
Donor risk index	26 (22–32)	26 (19–40)	0.881
**Allograft Reperfusion Performance**			
*Well*	5 (27.8%)	10 (40%)	0.342
*Acceptable*	8 (44.4%)	10 (40%)	
*Moderate*	5 (27.8%)	3 (12%)	
*Poor*	0 (0%)	2 (8%)	
Cold ischemia time in min	545 (350–1140)	600 (360–900)	0.857
Warm ischemia time in min	51 (30–68)	45 (20–90)	0.596

CMVIg, anti-CMV hyperimmunoglobulin; LT, liver transplantation; CMV, cytomegalovirus; MELD, model of end-stage liver disease; ICU, intensive care unit; ACLF, acute-on-chronic liver failure; CLIF-C, chronic liver failure consortium; OF, organ failure; CRP, C-reactive protein; PCT, procalcitonin; IL-6, interleukin-6.

**Table 2:** Post-LT outcome at 3 months.

	Non-CMVIg (*n* = 18)	CMVIg (*n* = 25)	*p* Value
Systemic bacterial infection	7 (38.9%)	9 (36%)	0.847
Intra-abdominal bacterial infection	11 (61.1%)	7 (28%)	0.030
CMV infection	4 (22.2%)	1 (4%)	0.066
EBV infection	3 (33.3%)	0 (0%)	0.034
Biopsy proven allograft rejection	4 (22.2%)	0 (0%)	0.005
Mortality	7 (38.9%)	2 (8%)	0.014
Death by septic MOF	5 (27.8%)	1 (4%)	0.026

CMVIg, anti-CMV hyperimmunoglobulin; CMV, cytomegalovirus; EBV, Epstein–Barr virus; MOF, multiorgan failure.

**Table 3:** Univariate prognostic factors of 3-month mortality.

Variables	Alive at 3 Months (*n* = 34)	Dead at 3 Months (*n* = 9)	*p* Value
Recipients‘ Age	58 (32–68)	61 (39–67)	0.278
Male sex	21 (61.7%)	7 (77.8%)	0.370
MELD at listing	30 (7–40)	15 (12–40)	0.019
MELD at LT	38 (30–40)	40 (31–40)	0.582
Δ MELD	9 (0–32)	16 (0–28)	0.047
Waiting time from registration in months	2.25 (0.1–36)	6 (1.5–25)	0.134
Waiting time from final admittance in days	13.5 (3–71)	26 (3–43)	0.229
Duration of pre-LT ICU stay in days	5 (2–16)	10 (2–42)	0.002
Ascites at LT	32 (94.1%)	9 (100%)	0.456
Bacterial infection at LT	15 (44.1%)	5 (55.6%)	0.541
Grade of ACLF (3 vs. 1 or 2) at LT	26 (76.5%)	5 (55.6%)	0.214
Number of pre-LT organ failures according CLIF-C	4 (1–6)	5 (2–6)	0.579
CLIF-C OF score at LT	15.5 (11–18)	16 (11–18)	0.935
Ventilation at LT	13 (38.2%)	5 (55.6%)	0.349
Vasopressors at LT	29 (85.3%)	7 (77.8%)	0.587
Renal replacement therapy at LT	18 (52.9%)	7 (77.8%)	0.179
Fatal triad at LT	7 (20.6%)	5 (55.6%)	0.038
Lactate level in mmol/L at LT	3 (0.9–5.6)	4.9 (1.9–10)	<0.001
CRP level in mg/dL at LT	3 (0.4–6)	5.6 (0.8–7.2)	0.013
PCT level in ng/mL at LT	1.5 (0.3–49.9)	3.6 (2–35.5)	0.264
IL-6 level in pg/mL at LT	19 (5–45)	25 (6–78)	0.003
Peak Tac level in ng/dL	10 (7–23.5)	14 (7–20)	0.169
Lack of CMVIg treatment	11 (32.4%)	7 (77.8%)	0.014
Donors’ Age	57 (18–75)	46 (27–71)	0.172
Donor body mass index	1.9 (1.07–2.50)	1.62 (1.08–2.15)	0.082
Donor risk index	26 (19–40)	25 (22–32)	0.447
Moderate/poor allograft reperfusion	10 (29.4%)	0 (0%)	0.063
Cold ischemia time in min	585 (350–900)	720 (450–1140)	0.009
Warm ischemia time in min	51.5 (20–70)	40 (300–90)	0.681

MELD, model of end-stage liver disease; LT, liver transplantation; ICU, intensive care unit; ACLF, acute-on-chronic liver failure; CLIF-C, chronic liver failure consortium; OF, organ failure; CRP, C-reactive protein; PCT, procalcitonin; IL-6, interleukin-6; CMVIg, anti-CMV hyperimmunoglobulin.

**Table 4:** Multivariate prognostic factors of 3-month mortality.

Variables	HR	CI 95%	*p* Value
Lactate level (> vs. ≤3 mmol/L)	34.63	2.383–503.198	0.009
CMVIg treatment (no vs. yes)	21.76	1.540–307.368	0.023
Cold ischemia time (> vs. ≤600 min)	11.49	0.966–136.786	0.053

HR, hazard ratio; CI, confidence interval; CMVIg, anti-CMV hyperimmunoglobulin.
